# The Effect of Accelerated Aging on Polylactide Containing Plant Extracts

**DOI:** 10.3390/polym11040575

**Published:** 2019-03-28

**Authors:** Krzysztof Moraczewski, Magdalena Stepczyńska, Rafał Malinowski, Tomasz Karasiewicz, Bartłomiej Jagodziński, Piotr Rytlewski

**Affiliations:** 1 Faculty of Mathematics, Physics & Technical Sciences, Kazimierz Wielki University, Chodkiewicza 30, 85-064 Bydgoszcz, Poland; m.stepczynska@ukw.edu.pl (M.S.); tomakara@ukw.edu.pl (T.K.); bar.jag@ukw.edu.pl (B.J.); prytlewski@ukw.edu.pl (P.R.); 2 Institute for Engineering of Polymer Materials and Dyes, Marii Skłodowskiej-Curie 55, 87-100 Toruń, Poland; malinowskirafal@gmail.com

**Keywords:** polylactide, polyphenols, plant extracts, accelerated aging

## Abstract

In this study, natural extracts of plant origin were used as anti-aging compounds of biodegradable polymers. Coffee (0.5–10 wt%), cocoa, or cinnamon extracts were added to the polylactide matrix. The obtained materials were subjected to an accelerated aging process (720, 1440, or 2160 h) at 45 °C and 70% relative humidity under continuous UV radiation. The effectiveness of the tested extracts was compared to a commercially available anti-aging compound, 2 wt% of butylated hydroxytoluene. Visual evaluation, scanning electron microscopy, melt flow rate, thermogravimetry, differential scanning calorimetry, tensile strength, and impact tensile tests were performed. We show that the use of smaller amounts of tested extracts is particularly advantageous, which do not adversely affect the properties of polylactide-based materials at low contents. At the same time, their effectiveness in stabilizing tested properties during the accelerated aging process is mostly comparable to or greater than the reference compound.

## 1. Introduction

Ageing is a term used in many branches of polymer science and engineering when the properties of the polymer change over a period of time [1]. Factors such as thermal, electrical, electrochemical aging, mechanical, environmental exposure, and others have a significant influence on the decrease in polymer material properties during their use.

There are three basic processes for polymer chain destruction, leading to the reduction of the polymer’s molecular weight including depolymerization, destruction, and degradation processes. Depolymerization reaction is the opposite of polymerization, and results in the thermal or chemical decomposition of the polymer to the monomer [2,3,4]. The destruction process is about the decomposition of polymer chains with the release of low molecular weight chemical compounds that differ from the monomer [5,6,7]. It can be caused by physical factors (heat, light radiation, and high energy radiation) or chemical (oxygen, acids, and bases). Degradation is the partial decomposition of the polymer, however, not to low molecular weight products but to fragments with higher molecular weight [8,9,10]. The factors that initiate degradation can be physical (stresses, heat, and high energy radiation) as well as chemical (oxygen and aggressive media).

Among the mentioned the degradation processes occur most frequently during processing and everyday use of polymer materials. The rate of degradation during processing as well as during service life depend on the chemical structure, presence of structural defects, impurities such as polymerization catalyst residues, the environment to which the polymer is exposed and the use of stabilizers. Degradation leads to structural changes that may be a result of physical or chemical processes [11]. The degradation process usually causes irreversible changes in the polymer, resulting from phenomena such as: crosslinking, thermal oxidation, chain cutting, or destruction. These phenomena occur in polymeric materials under the influence of long-lasting external factors, such as: heat, oxygen, ozone, light radiation, high energy radiation, UV radiation, chemical substances, including water and steam, as well as mechanical stresses [12,13,14,15,16].

All these factors can act on the material separately or simultaneously. The number of interacting factors and their intensity depend on climatic zone, season of the year and environmental pollution. Most often these interactions are synergistic and result in interactions between particular factors. The physicochemical properties, such as molecular weight, chain structure, glass transition temperature, melting point, crystallinity, surface area of the product, hydrophilicity, type and content of fillers, also influence the course and speed of polymeric materials’ degradation process [12,17,18].

Changes in the properties of material used outdoors as a result of interaction with climatic factors are called atmospheric aging or weathering. During atmospheric aging, essentially two processes dominate causing changes in the chemical structure of the polymer, i.e., photooxidation and thermooxidation [9]. Photooxidation is a process caused mainly by UV radiation in the presence of oxygen, and occurs mainly on the surface of the exposed material. Thermooxidation takes place under the influence of heat and oxygen. It depends on the material characteristics, oxygen pressure, temperature, process initiation rate, and sample thickness. The influence of various variable parameters makes the mechanism of both mentioned degradation processes complex and includes many parallel and subsequent reactions.

As in the case of “classic” polymers obtained from petroleum, biodegradable polymers also need to have suitable resistance to external factors. The most common method to improve stability of polymer materials is the use of specific chemicals known as anti-aging compounds. Anti-aging compounds added to the polymer matrix improve materials’ resistance to elevated temperatures, UV radiation or chemical substances [19,20,21].

Specifications of biodegradable polymers impose some restrictions on chemical compounds that can be used as anti-aging compounds. These compounds should be non-toxic, non-volatile, do not migrate to polymer surface or pollute the degradation environment or adversely affect biodegradation process. It is important that the used anti-aging compounds allow contact of manufactured products with food, as one of the main applications of biodegradable polymers is packaging (food foil, disposable tableware) [22,23]. Anti-aging compounds for applications in biodegradable polymers should also be environmentally friendly and not pollute the degradation medium.

The best solution would be to use natural substances of vegetable origin. First of all, their impact on the environment would be significantly smaller compared to synthetic compounds. Secondly, their natural origin and short degradation times would not cause excessive extension of degradation time of biodegradable polymers. Natural origin compounds are generally easily available, do not require special processes of chemical synthesis, and thus cheap. Due to the composition and chemical structure, substances of vegetable origin containing natural polyphenols may be a very promising anti-aging compounds. Plant polyphenolic have been shown to act as strong antioxidants in various systems and their multiple biological actions have been extensively reviewed [24,25,26,27,28,29].

Research carried out so far by the authors showed that coffee can be used as an anti-aging compound of polymeric materials [30]. However, it seems advisable to extend the conducted research with new substances of vegetable origin and the possibility of using them to stabilize biodegradable polymers.

Many types of biodegradable polymers have been developed, but due to good processing and exploitation properties, polylactide (PLA) is of great interest [31,32,33,34,35,36]. PLA is a biodegradable and biocompatible polyester that can be produced from renewable resources. It is synthesized from lactic acid obtained from the fermentation of cornstarch or sugar cane. This polymer is widely used in the production of food packaging, textiles, tissue engineering, and the production of a cell culture medium.

The scientific aim of the study was to determine the possibility of using natural substances as anti-aging compounds of biodegradable polymers. The use of natural anti-aging compounds will improve the resistance of biodegradable polymers to external factors causing the aging of these polymers, without negative effect on their biodegradation process during composting and the possibility of using these polymers in the production of food packaging. The first stage of research was to determine the characteristics of polymeric materials containing tested substances [37,38].

The paper presents the results of selected properties of PLA-containing natural anti-aging compounds and subjected to the process of accelerated aging. Samples containing 0.5–10 wt% of plant extracts were tested. Selected properties of PLA-containing natural anti-aging compounds subjected to an accelerated aging process were investigated and compared with samples containing a commercially used anti-aging compound.

## 2. Materials and Methods

Polylactide (PLA) type 2003D (Cargill Dow LLC, Minnetonka, MN, USA) with a mass melt flow rate (MFR) of 6 g/10 min (210 °C, 2.16 kg) and density (ρ) of 1.24 g/cm^3^ was used as materials matrix. Three natural compounds of vegetable origin were used: coffee extract, cocoa extract, and cinnamon extract (Agrema Sp. z o.o., Wrocław, Poland). According to the data sheets, the coffee extract contained 45 wt% of polyphenols, while extracts of cocoa and cinnamon contained 5 wt% of polyphenols. The main polyphenols in coffee, cocoa, and cinnamon extracts were chlorogenic acid, flavonoids, and phenolic acids, respectively.

Properties of the obtained materials were compared with material containing a commonly used synthetic anti-aging compound. As a reference compound butylated hydroxytoluene (BHT) (Sigma-Aldrich, Saint Louis, MO, USA) was used with a ρ of 1.04 g/cm^3^. This compound is commonly used as an anti-aging additive for various polymers [39,40,41].

The test materials were produced in two stages. In first stage the amount of 0.5, 1, 3, 5, or 10 wt% of natural anti-aging compounds were added to the PLA matrix, to give samples designated as P0.5, P1, P3, P5, and P10 respectively. Most often, anti-aging compounds are used in an amount not exceeding a few percent. In the case of tested natural anti-aging compounds, larger amounts of extracts were also used. This is due to the fact that, unlike synthetic anti-aging compounds, only part of the extract is an active substance with anti-aging properties. A PLA granulate containing 2 wt% BHT was also produced, which was a reference material designated as R sample. The sample of pure PLA was designated as P sample. In the first stage extrusion process, a co-rotating twin-screw extruder type BTSK 20/40D (Bühler, Germany) was used. The temperatures of the individual zones of the extruder and head were 170, 175, 180, 180, and 180 °C respectively. Intensive mixing screws with additionally retracting and kneading segments have been applied. The material after leaving the head was cooled by the air stream and then granulated.

In the second stage, the films were produced on a single-screw extruder Plasti-Corder Lab Station (Brabender, Duisburg, Germany). The temperatures of cylinder zones were 165, 175, and 168 °C. The extrusion process used a flat-faced head with a mouthpiece width of 170 mm. The head temperature was 158 °C. A 4:1 screw with a mixing tip was used. The film was poured onto thermostatic rolls at a temperature of about 30 °C. The thickness of the obtained film was approx. 0.5 mm.

The accelerated aging was performed in climatic chamber CCK 40/300NG (Dycometal, Viladecans, Spain) at a temperature of 45 °C, 70% relative humidity, and UV irradiation for 720, 1440, or 2160 h. The chamber has 8 fluorescent lamps (Philips Super Actinica TL 60W/10-R ISL, Amsterdam, Holland) with 350–400 nm wavelength. Samples were placed directly under the lamps, so that the sample distance to the lamp was as small as possible. The samples were arranged perpendicular to the UV source.

The melt flow rate (MFR) was determined according to the procedure specified in [42], using a MP 600 plastometer (Tinius Olsen, Horsham, PA, USA). The MFR (190 °C, 2.16 kg) was determined using 12 individual samples. The final values of MFR were calculated as the arithmetic mean of 10 results (two extreme value were neglected).

The tensile tests were carried out according to the procedure specified in [43]. The extension rate for testing each sample was 50 mm/min, determined using 12 individual samples. The tests were carried out at room temperature calibrated to 21 °C. The final values were calculated as the arithmetic means of 10 results, two extreme ones being neglected.

The tensile-impact strength (a_tU_) tests were carried out according to the procedure specified in [44], using the IMPASS-15 (ATS FAAR, Novegro-Tregarezzo, Italy) impact hammer. Hammer energy was 4 J. The hammer speed at the moment of breaking the sample was 2.9 m/s. The tests were carried out at room temperature calibrated to 21 °C. The impact tensile strengths were determined using 12 individual samples. The final values were calculated as the arithmetic means of 10 results, two extreme ones being neglected.

The differential scanning calorimetry (DSC) measurements were carried out in a nitrogen atmosphere, according to the procedure described in [45,46,47] using a Q500 differential scanning calorimeter (TA Instruments, New Castle, DE, USA). Samples of ~7 mg were used. The measurement temperature range was 0–180 °C. The DSC curves were recorded in three stages: first heating, cooling, and second heating. The rate of temperature change was 10 °C/min.

The thermogravimetric (TG) measurements were performed in a nitrogen atmosphere, according to the procedure described in [48] using a Q200 thermogravimetric analyzer (TA Instruments, New Castle, DE, USA). The mass of individual samples was ~22.5 mg. The TGA measurements were carried out at 20–600 °C and with a heating rate of 10 °C/min.

Figures in the article present the results of selected samples only. Only those samples are shown for which the values and observed changes were closest to the reference sample at the lowest possible level of the tested anti-aging compounds. The results of all samples obtained using the applied research methods are presented in the tables.

## 3. Results and Discussion

### 3.1. Visual Evaluation

**Figure 1:**
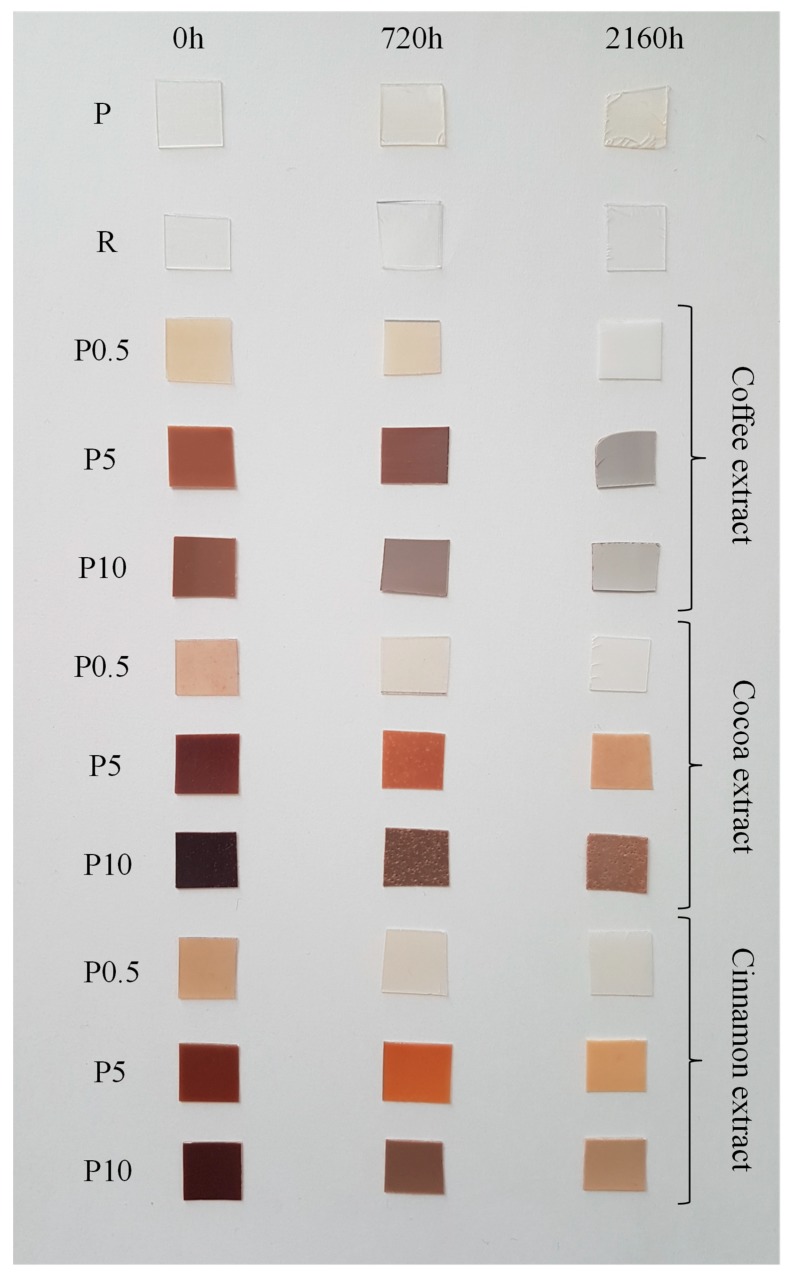
Photos of selected samples before and after the aging process.

The change in color of tested materials is the first easily perceptible effect of the adverse impact of external factors. In the outdoor applications changes in the color of the polymeric components can be a significant problem. Changes in the color of the specimens were evaluated by comparing their photographic images after each accelerated weathering period. Photos of selected un-aged and aged (for 720 or 2160 h) samples are shown in Figure 1.

Un-aged P and R samples were colorless with a high degree of transparency. High transparency of samples was due to the advantage of the amorphous phase and the low content of crystalline phase. Un-aged samples containing tested extracts were brown. However, color intensity and transparency was dependent on extract content. Samples containing 0.5 wt% of extracts were transparent with a slightly brown color. The samples became darker and less transparent as the extract content increased. After adding 5 wt% of the extracts the sample became opaque with an intense brown color. The increase in the content of extracts resulted in further darkening of the color.

Color and transparency are important parameters that are taken into account when using polymeric materials for packaging production. A clearly visible level of reduction of these parameters appeared only above 3% of extracts. Below this content, the samples were not colorless, but transparency was sufficient for most applications. It should be mentioned, however, that the thickness of the material used in the tests was quite large (0.5 mm) as for films used in the packaging industry. In most cases, foils up to a thickness of 0.1 mm are used. In the case of thinner films, transparency should be much better even with higher extract contents. However, even lack of transparency at higher extract content does not eliminate these materials from applications to products with a short life span. They can be successfully used in the production of biodegradable disposable dishes and cutlery, straws, ear buds, sticks, garbage bags, etc. In all these applications, transparency is not required, and the possibility of biodegradation of such products, which very often are a heavy burden for environment, will be a big advantage.

The P sample becomes slightly yellow as a result of aging. The change in color may be the result of chemical or physical reactions taking place during this process. These reactions cause the loss of materials primary properties and the color change is the first visible sign of that phenomena. The transparency of P sample does not change after aging, which suggests that there were no visible changes in the structure of the surface or crystal structure that would lead to clouding of P sample. In the case of samples with low extract content, the color disappeared completely with a simultaneous decrease in the transparency after 2160 h of aging. The changes in transparency were probably due to tarnishing on the surface of polymers (rather than a change in the degree of crystallinity, which was confirmed by later DSC studies).

Aging of samples with higher extracts content led to color brightening. Particularly large changes were observed for samples containing more than 5 wt% of coffee extract, where the color changed from dark brown to light gray. Intensive color change of samples containing coffee extract may suggest that the compounds in this extract strongly interact with UV radiation. UV radiation causes decomposition of these compounds, which leads to a complete disappearance of color. No major changes in color and transparency due to accelerated aging were observed in the case of the R sample.

### 3.2. Melt Flow Rate

The mass melt flow rate (MFR) of processed, pure PLA was 5.6 g/10 min. MFR values for the R sample were slightly lower and reached 4.7 g/10 min. MFR results for all samples are presented in Table 1.

A very large MFR increase in un-aged samples was observed after adding the coffee extract to PLA. Adding 0.5 wt% of the extract caused a threefold increase in MFR value, while the addition of 10 wt% caused a nearly thirteen fold increase in this value. A significantly smaller increase in MFR was observed for the cocoa extract. A two-fold increase was observed after adding 3 wt% of cocoa extract. Increasing the cocoa extract to 10 wt% resulted in a fourfold increase in MFR compared to the pure PLA sample. The smallest changes in MFR were observed in the case of cinnamon extract. Up to 3 wt% of the extract’s MFR value was close to that of pure PLA. Further increase in the extract concentration caused only a slight increase in MFR. The MFR value obtained for material containing 10 wt% cinnamon extract was only about one and a half times the value of pure PLA.

**Figure 2:**
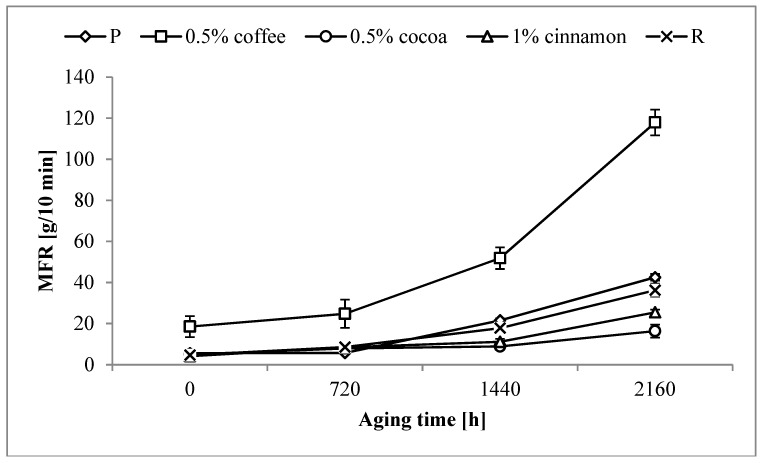
Changes in the melt flow rate (MFR) of selected samples as a function of aging time.

As a result of aging the MFR value of pure polymer increased (Figure 2). After the longest aging time, MFR of the P sample increased almost eight times. The addition of the reference anti-aging compound did not reduce this growth. MFR of the R sample after the longest aging time was also almost eight-fold higher than the un-aged sample.

Much larger changes were observed in samples containing coffee extracts. Even in the sample containing 0.5 wt% of the extract, the MFR value after the aging process increased significantly. Although the relative increase for samples containing coffee extract was smaller than for samples P and R (MFR increased from two to six times) the values after the aging process were very high.

A positive effect on MFR values was observed for samples containing cocoa and cinnamon extracts, especially at lower levels of these extracts. In most cases the MFR values were close to or lower than the values obtained for P and R samples. The most stable MFR during aging was obtained for sample containing 0.5 wt% cocoa extracts. The value of MFR did not increase significantly up to 1440 h of aging. After the longest aging time the MFR of this sample increased threefold, but it was still almost three times lower than the value for sample P, and twice lower than the value of the R sample. In the case of cinnamon extract, the best effects were obtained for sample containing 1 wt% of the extract. The overall increase in MFR was higher than in the case of samples with similar amounts of cocoa extracts, but the value obtained after the longest aging time was lower than the values obtained for P and R samples.

### 3.3. Thermogravimetric Analysis

In thermogravimetric analysis, the temperature T_5%_ corresponding to the temperature of 5% mass loss was determined. This value was assumed as the beginning of material’s thermal degradation. T_5%_ values of tested samples are presented in Table 2.

All tested extracts caused a decrease in T_5%_ for un-aged samples. The values obtained for samples containing proposed extracts, however, still were much higher than the typical temperatures of PLA products’ application. Especially in the case of cinnamon extract the thermal resistance was only slightly lower than the thermal resistance of PLA up to 10 wt% of extract. Particularly large changes were observed in the case of coffee extract, what was probably related to far higher polyphenol content (45 wt% vs. 5 wt%). Extracts of cocoa and cinnamon, due to lower concentration of polyphenols, had much less influence on decrease of thermal resistance. To confirm this assumption the straight comparison between coffee and the other extracts was done with about 1/10 ratio between extracts. We observed that the values for T_5%_ of 1 wt% coffee extract and 10 wt% cocoa or cinnamon extracts were very close.

**Figure 3:**
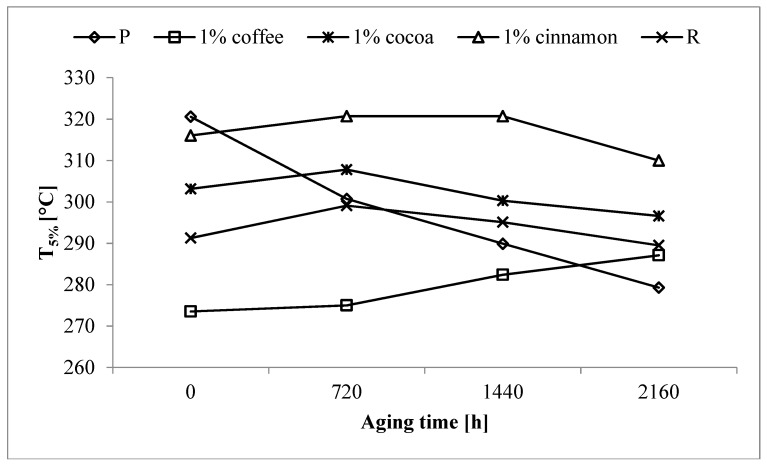
Changes in the temperature of 5% of mass loss (T_5%_) of selected samples as a function of aging time.

Aging of pure PLA caused a decrease in T_5%_ of the P sample by 41 °C (Figure 3). The aging process probably led to polymer destruction involving the decomposition of polymer chains and the formation of low molecular weight compounds. The low molecular weight compounds have lower thermal stability and should be degraded and/or volatilized more easily, thus lowering the T_5%_ value.

The destruction process may be caused by physical factors such as heat and UV radiation, which were used in the process of accelerated aging [49,50,51,52,53,54]. Adding a commercial anti-aging compound did not cause any change in the T_5%_ value of the R sample after the longest aging time, which remained close to the value of the un-aged sample. This suggests that adding this compound to PLA matrix reduces the adverse effects of physical factors and limits the processes of macromolecule destruction. Similar effects were observed after adding other tested extracts. Taking into account the lowest initial drop in T_5%_ and its stability during the aging process, the use of cocoa and cinnamon extracts proved to be the most effective. Despite the fact that the T_5%_ value of aged samples containing coffee extract remained at a similar level during aging, large decrease in the initial value of T_5%_ favors the other two extracts.

For samples containing cocoa extracts, the most stable value of T_5%_, with the lowest possible initial decrease was obtained for the sample containing 1 wt% of the extract. After the longest aging time, the T_5%_ value of this sample was only ~7 °C lower than the value obtained for the un-aged sample. A similar low decrease was observed in the case of the sample containing 1 wt% of cinnamon extracts. It should be noted however, that the T_5%_ values obtained for this sample were higher than those for the sample containing the same amount of cocoa extracts. Furthermore, other aged samples containing cinnamon extracts were characterized by higher values than the corresponding samples containing the cocoa extract.

Studies show that the use of small amounts of extracts have a positive effect on the thermal stability of samples subjected to accelerated aging. Adding tested extracts to polymer matrices suppressed the decrease in temperature at the beginning of thermal degradation, which was significant in the case of the pure PLA sample. The effectiveness of these extracts was comparable to the effectiveness of the reference anti-aging compound.

### 3.4. Differential Scanning Calorimetry

Analysis of DSC results was based on the first and second heating run. The first heating determined the influence of the accelerated aging process on selected thermal properties of the samples. The second heat determined the effect of accelerated aging on the structure of the investigated materials after thermal history removal. Glass transition temperatures (T_g1_ and T_g2_), cold crystallization temperature (T_cc1_ and T_cc2_), melting temperatures (T_m1_ and T_m2_), and degree of crystallinity (X_c1_ and X_c2_) were determined from the each heating run of DSC thermogram. The X_c_ values were calculated based on Equation (1) assuming that the value of enthalpy change of 100% crystalline PLA (ΔH_m100%_) is 93 J/g [55].

(1) $$X_{c}=\left(\frac{\Delta H_{m}-\Delta H_{\mathrm{cc}}}{\Delta H_{m100\%}}\right)\cdot 100\%$$

The obtained results are presented in Table 3.

Based on the obtained results, it can be concluded that the addition of extracts to the PLA matrix did not cause a large changes in the T_g_ values of un-aged samples during both the 1st and the 2nd heating cycles. Only in the case of samples containing 3 wt% or more coffee extracts, larger changes in T_g1_ and T_g2_ were observed. A clear decrease in T_g_ was observed for the R sample. The addition of a commercial anti-aging compound resulted in a decrease in T_g1_ by 1.5 °C and T_g2_ by 3 °C compared to the values of pure PLA.

**Figure 4:**
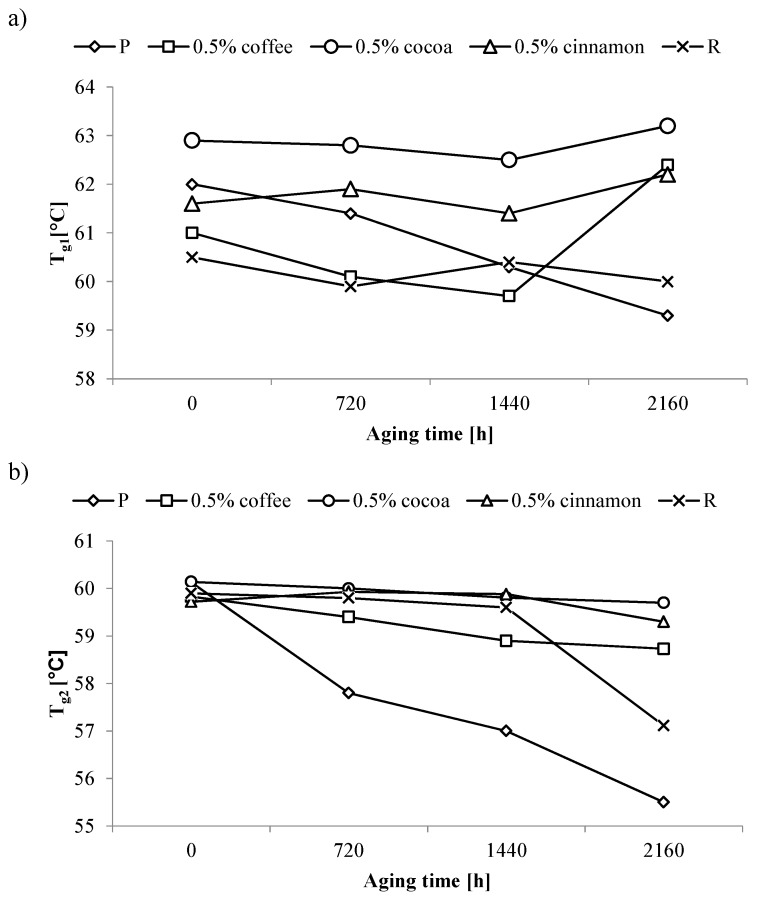
Changes in the glass transition temperature (T_g_) of selected samples as a function of aging time; (**a**) first heating and (**b**) second heating.

The T_g1_ values of pure PLA obtained from the first heat DSC curve showed approximately linear decrease with increasing aging time. After 2160 h of accelerated aging, the value obtained for the P sample was lower by 2.7 °C (Figure 4a). Adding a reference anti-aging compound caused a decrease in the T_g1_ value of the un-aged R sample, but this value maintained at a similar level regardless of the aging time. Similar stability was obtained for samples containing just 0.5 wt% of cocoa extracts or cinnamon, for which T_g1_ values were even higher than those for the R sample. A different curve characteristic was observed for the sample containing 0.5 wt% coffee extracts. Initially, a decrease in T_g1_ was obtained along with an increase in aging time. Then after the longest period of aging a rapid increase in T_g1_ was observed. A similar effect was present in samples containing larger amounts of coffee extracts.

A significantly greater influence of the aging process on T_g_ values for pure PLA was observed in the case of the second heating cycle (Figure 4b). T_g2_ values of the P sample after the longest aging period was lower by 4.5 °C compared to the value obtained for the un-aged sample. T_g2_ values for the R sample were constant up to 1440 h of aging. Longer aging time resulted in a rapid decrease in the T_g2_ values by 2.8 °C from those obtained for the un-aged sample. On the other hand adding tested anti-aging compounds to the PLA matrix caused T_g2_ values to remain constant throughout the accelerated aging process. High stability of T_g2_ values was already obtained for 0.5 wt% of the tested extracts.

The decrease in the T_g_ values of the P sample observed as a result of accelerated aging may be the result of two phenomena. First, low molecular weight compounds arise in the process of macromolecule destruction. These compounds have plasticizing properties lowering the glass transition temperature of polymeric materials. Second, it may be due to the shortening of macromolecules caused by breaking of polymer main chain. The resulting shorter macromolecules are characterized by lower glass transition temperatures [56].

The aging of polymeric materials is a complex process so it cannot be clearly indicated, which of the corrosion mechanisms prevails. Therefore it is correct to assume that lowering T_g_ results from both described phenomena in a difficult to determine ratio. Nevertheless it is important to note that the low content of tested extracts stop this process in a more effective way than a commercial anti-aging compound. The compounds contained in the extracts limit the phenomenon of the formation of small molecule compounds and/or the cracking of the macromolecule chain, which results in the lack of lowering of T_g_.

**Figure 5:**
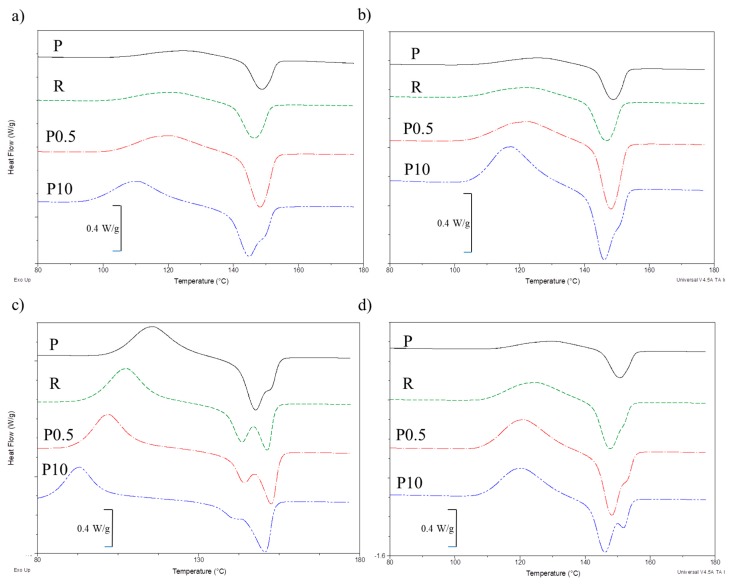
DSC curves of selected samples; (**a**) first heating of un-aged samples, (**b**) second heating of un-aged samples, (**c**) first heating of samples aged for 2160 h, and (**d**) second heating of samples aged for 2160 h.

Figure 5 shows the DSC curves of the P and R samples as well as samples containing 0.5 and 10 wt% of coffee extract before and after aging process. The curves of samples containing cocoa extracts or cinnamon extracts were similar, therefore for the readability of the graph, only one type of extract was presented.

DSC curves of first and second heating scan of un-aged samples were very similar. Therefore, a further description for un-aged samples was applied to both runs. The T_cc_ and T_m_ temperatures of the P sample had similar values in both cases. In the case of the R sample, no significant changes between the two heating curves were observed. The obtained T_cc_ and T_m_ values were however ~4 °C lower than those for the P sample. No significant differences between the first and second heating curves were observed in the case of un-aged samples containing tested extracts. However, the quantity and type of extracts affected the obtained T_cc_ and T_m_ values of individual samples and the DSC curve characteristic.

With the increase in coffee and cocoa extracts, T_cc_ peak maximum shifted towards the lower values. The observed difference in T_cc_ between the extreme contents of the extracts was ~9 °C. As a result of the increase in the content of these extracts, the intensity of the cold crystallization phenomena also changed. Cold crystallization peaks with a larger area were observed for the un-aged samples with higher extract content. The changes were probably caused by the nucleating properties of coffee and cocoa extracts, which facilitate the process of crystallization. However, a significant difference in T_cc_ and the intensity of the cold crystallization process were not observed for the cinnamon extracts, suggesting that its nucleating capabilities were significantly smaller than the other extracts.

Changes in cold crystallization were accompanied by changes in the melting process. However, differences in T_m_ values for un-aged samples were not as large as in the case of T_cc_, which suggests that the resulting crystalline structure for individual samples were similar. Along with the increase in the content of extracts, T_m_ values only slightly moved towards lower temperatures. The largest decrease was observed for coffee extracts. However, it was only ~3 °C.

Changes in the DSC curves between first and second heating were observed after the accelerated aging process. Especially in the case of the first heating, the obtained values of determined parameters changed significantly in comparison to those of the un-aged samples.

Aging of the P sample resulted in a clear decrease in the T_cc1_ value. After 2160 h of aging, the value decreased by ~10 °C. A slightly larger decrease in T_cc1_ of ~14 °C was observed for the R sample. In the case of the tested extracts, T_cc1_ shifted towards lower values along with the increase in aging time. The lowest values were obtained for coffee extracts, and the highest for cinnamon extracts. In most cases, the differences between the values of un-aged and 2160 h aged samples remained at a similar level regardless of the content of individual extracts. After the aging process, no significant changes in the intensity of the cold crystallization peak were observed. Peak areas for samples with different contents of individual extracts were almost identical.

The phenomenon of decrease in cold crystallization temperature as a result of aging, is characteristic of PLA [57,58]. The most probable explanation for such accelerated crystallization is that the mesomorphic phase (the intermediate inhomogeneous phase between crystalline phase and homogeneous amorphous phase) formed during PLA aging facilitates the formation of crystallite nuclei, resulting in the rapid crystallization during DSC heating. Increasing the number of nucleation centers in the case of samples containing the tested extracts further intensified this phenomenon.

The DSC first heating curve in the area of the melting peak also changed as a result of accelerated aging. After 2160 h of aging, more or less bimodal melting peaks were observed. The bimodal peaks were present in both P and R samples, as well as the samples containing all tested extracts. In addition to the main peak of the melting phenomena, where T_m1_ was close (cinnamon extract) or higher (coffee and cocoa extract) to the value of the un-aged sample, a second arm appeared with the maximum shifted towards the lower temperatures. There are two possible ways to explain the phenomenon of a bimodal melting peak. The first is due to the fact that aged PLA very often exhibits a double melting behavior associated with the stable pseudoorthorhombic structure melting at higher temperatures (α-form) and the orthorhombic (β-form) that melts at a lower temperatures [59]. There is also a second possibility for the occurrence of a double melting peak. The first melting peak is sometimes attributed to the melting of more organized crystals when defective crystals reorganize simultaneously then melt at higher temperatures, providing the second peak. However, this phenomenon is more common during isothermal PLA crystallization, where the crystalline phase is formed when the material is held at a constant temperature [60]. Since the absolute values of cold crystallization and melting enthalpy are almost the same we can assume that in our case, the first phenomenon is responsible for the occurrence of a bimodal peak. T_m1_ values for the additional peak and its surface area decreased with increasing content of the extracts. The peak with a higher T_m1_ value was more and more dominant implying that the small and less perfect crystals disappear as the content of extracts increases.

After erasing the thermal history the DSC curves of the second heating of the aged samples resembled the curves obtained for the un-aged samples. T_cc2_ values were very close to the values obtained for un-aged samples, regardless of the aging time. Some differences, however, were observed in the intensity of the cold crystallization peak. After 2160 h, the peaks were larger than those for un-aged samples containing the same extract content. Furthermore, T_m2_ values for the aged samples were very close to those of the un-aged samples. A clear bimodal melting peak with the dominating arm at lower temperatures was observed only for samples containing 10 wt% of the extracts.

The extracts and the process of accelerated aging had no major influence on the degree of the tested materials’ crystallinity. Most samples were almost completely amorphous, since the absolute values of the cold crystallization and melting enthalpy were almost the same. PLA has very slow crystallization rate. Thus due to the very fast cooling rate during processing and the relatively fast cooling rate applied (10 °C/min), macromolecular chains of PLA have no sufficient time to crystallize [61]. The values of X_c1_ and X_c2_ of pure PLA were small; 0.3 and 0.1%, respectively. The addition of a reference anti-aging compound resulted in an increase in the value of X_c1_ to 2.4%. In most cases the X_c1_ values of the un-aged samples increased with increasing content of extracts. Observed changes were, however, very small and could be the result of measurement error. A significant increase was observed only at the highest content of extracts.

Major changes in value were not observed for X_c2_. For the R sample, and samples containing the tested extracts, changes in the X_c2_ value of the un-aged samples were negligible. Furthermore, after the samples’ accelerated aging, the values of X_c1_ and X_c2_ did not change significantly. Only in the case of samples containing 10% of extracts, greater changes were observed, but without a clearly visible trend. It can therefore be concluded that adding test extracts in an amount below 5% does not affect the tested materials’ degree of crystallinity.

### 3.5. Tensile Tests

The tensile strength (σ_M_) and tensile strain at break (ε_B_) were determined via a tensile strength test. The value of σ_M_ of P sample was 62 MPa, while the R sample was lower by ~7 MPa. The addition of extracts caused a slight decrease in mechanical properties determined in tensile test. Only in the case of material containing 10 wt% of coffee extracts, a significant decrease in σ_M_ values was observed. However, it should be noted that the values obtained for the tested materials were in most cases higher than the values obtained for the R sample. Results of the tensile test are presented in Table 4.

For almost all samples, the aging process caused an initial increase in tensile strength. The increase in σ_M_ was particularly evident for the R sample, and samples containing smaller extract content. The smaller the content of extracts in the examined material, the higher the increase in the value of σ_M_ caused by aging was observed. The increase in the value of σ_M_ was probably caused by the rearrangement of polymer macromolecules due to prolonged exposure to elevated temperature, which led to improved mechanical properties. At higher extract contents, the rearrangement process could be hindered, hence smaller changes in tensile strength were observed.

**Figure 6:**
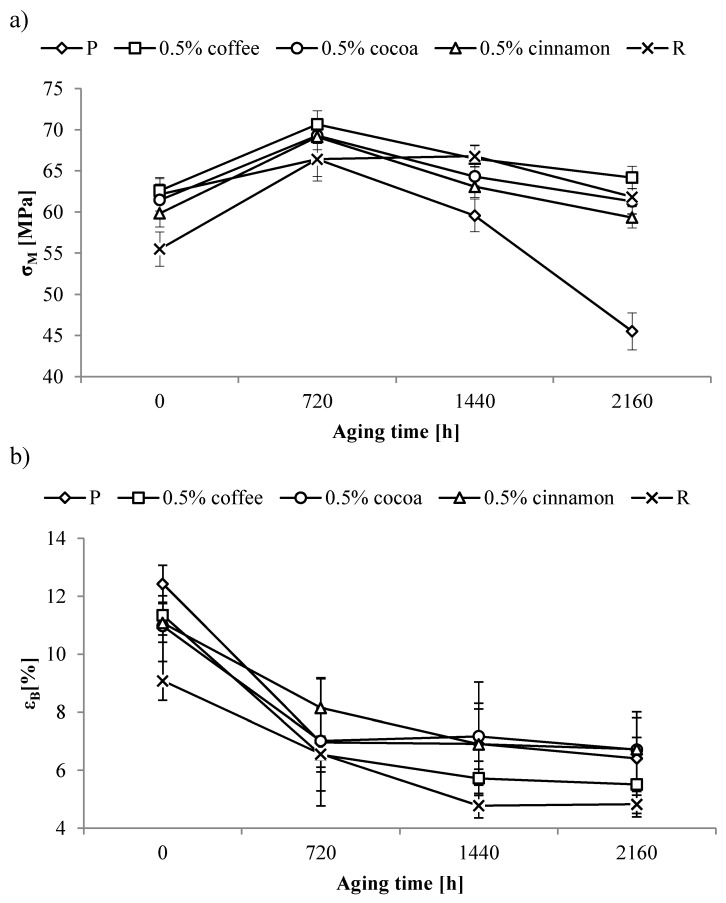
Changes in (**a**) tensile strength (σ_M_) and (**b**) tensile strain (ε_B_) of selected samples as a function of aging time.

After a longer aging time, the tensile strength of the tested samples decreased. The largest decrease was observed for pure PLA. σ_M_ values for the P sample after 2160 h of aging decreased by ~17 MPa from the un-aged sample reaching 45.5 MPa (Figure 6a). A drop in tensile strength was due to macromolecular chain scission induced by exposure to accelerated aging. The decrease in σ_M_ values for the R sample was much smaller (~5 MPa), and the final value after the aging was higher by ~6 MPa from the value of the un-aged sample. It follows that the reference anti-aging compound limited the phenomenon of the main chain scission, thereby limiting the loss of mechanical strength.

A similar effect as in the case of the R sample was observed for samples containing small amounts of the tested extracts. Adding 0.5 wt% of coffee, cocoa, or cinnamon extracts limited the decrease in tensile strength in a similar range to the reference anti-aging compound, which may suggest that these compounds also limit the scission of the macromolecule polymer chain. The obtained values were higher than the values obtained for the P sample, and σ_M_ values after aging were close to those of the R sample. However, increasing the content of extracts aggravated the tensile strength of the samples subjected to aging. At the highest extracts content, obtained values were similar or worse than the values obtained for the P sample.

Changes in tensile strength of the un-aged samples were accompanied by changes in strain at break. The addition of the reference anti-aging compound to PLA matrix caused a decrease in the ε_B_ value of the R sample by 3.3 units in comparison to the P sample. Furthermore, adding tested extracts to the PLA matrix caused a decrease in ε_B_. ε_B_ decreased with the increase in extract content, a phenomenon typical for powder fillers. The quantity of change was affected by the type of extract. The smallest effect of the extract content on ε_B_ was observed for samples containing coffee extract, wherein only the addition of 10 wt% resulted in a large decrease. In contrast, the most pronounced effect was observed for the cocoa extract, where already 3 wt% of the extract caused a significant decrease in ε_B_.

As a result of the accelerated aging, the strain at break values were reduced. In the case of the pure PLA sample, the greatest decrease was observed after 720 h of aging (Figure 6b). The ε_B_ value of the P sample decreased from ~13% to 7%. A smaller decrease was observed for the R sample, where the ε_B_ value after 720 h of aging decreased from 9% to 7%.

In the case of samples containing the tested extracts, the course of ε_B_ changes after accelerated aging depended on the type and content of the extract. A particularly large decrease after 720 h of aging, similar to that for sample P, was observed for samples with a lower content of cocoa or cinnamon extracts (Figure 6b), where ε_B_ decreased from 11% to 7% and from 11% to 8%, respectively. In the case of coffee extracts, the similar course of changes was maintained up to 5 wt% of extract, where a decrease from ~11% to 7% was observed.

A further increase in aging time did not significantly affect changes in the ε_B_ values both for the P sample and the samples containing tested extracts. Regardless of the type and content of the extract, ε_B_ values were maintained at a similar level. Only in the case of the R sample, a larger decrease was observed after 1440 h of aging, where the value decreased from 6.6% to 4.8% and remained at this level.

A large initial decrease in strain at break due to the aging process can be explained by the arrangement of polymer macromolecules due to prolonged exposure to high temperatures. As a result of this process, the material becomes more rigid, resulting in less deformation during the tensile test.

Research shows that the use of small amounts of extracts has a positive effect on the stability of tensile strength of samples undergoing an accelerated aging process. The addition of test extracts to the matrix suppressed the decrease in tensile strength observed in case of the pure PLA sample. The effectiveness of these extracts was better than the effectiveness of the reference anti-aging compound. The similar nature of the change of strain at break for pure PLA samples and samples containing the tested extracts shows that they do not significantly affect this parameter as a result of the accelerated aging process.

### 3.6. Impact Tensile Strengh

Impact tensile strength (a_tU_) of the P sample was 140 kJ/m^2^. The addition of a reference anti-aging compound to the PLA matrix resulted in an increase of this value to 145 kJ/m^2^. The tested extracts caused changes in a_tU_ values, whose character was dependent on the type of extract. The results of impact tensile strength of all samples are presented in the Table 5.

In the case of un-aged samples with coffee extracts, only after the addition of 10 wt% was a significant decrease in a_tU_ observed. The a_tU_ was ~14 MPa lower than that for the P sample. For the cocoa extract, a significant decrease of approx. 9 MPa followed the addition of 3 wt% of the extract. In turn, a significant decrease occurred after adding only 0.5 wt% of cinnamon extracts. The values of samples containing cinnamon extract were lower by 6–12 MPa than the P sample value. It should be noted, however, that the values of a_tU_ of all samples containing tested extracts were lower than the value obtained for the R sample.

**Figure 7:**
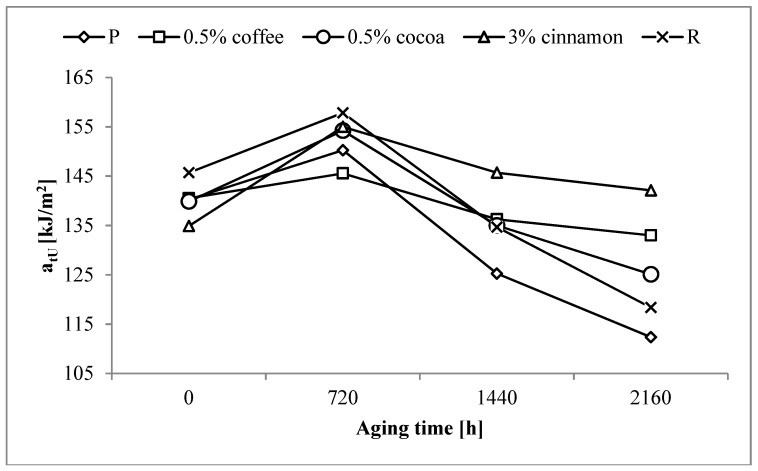
Changes in the impact tensile strength (a_tU_) of selected samples as a function of aging time.

As in the case of tensile strength accelerated aging caused an initial increase in a_tU_ for most samples. It was also probably due to the reorganization of the polymer macromolecules. a_tU_ values for P and R samples after 720 h of aging increased by 10 and 12 MPa, respectively (Figure 7). In the case of studied extracts the rate of increases varied depending on the type and content of the extract, however, without a noticeable trend of changes.

After the initial increase, a_tU_ decreased when aging time was longer. The lowest value was observed for the pure PLA. a_tU_ for the P sample after 2160 h of aging decreased by ~28 MPa with respect to the un-aged sample reaching 112.4 MPa. Decrease in the a_tU_ value of the R sample was practically the same as that of the P sample, however, the final strength after the aging process was about 6 MPa higher than the aged P sample.

Significantly lower decreases in a_tU_ were observed for samples containing tested extracts. In the case of coffee and cocoa extracts, the most favorable results with the lowest possible content of extracts were obtained for samples containing 0.5 wt%. The total decrease in the a_tU_ values was ~8 MPa for the coffee extract and ~15 MPa for the cocoa extract. The values of a_tU_ after 2160 h of aging reached 133.0 MPa and 125.1 MPa, respectively, and were higher than the value obtained for the aged R sample. In the case of cinnamon extract, the most favorable results were obtained with a higher extract content. The most stable and close to the value obtained for the un-aged P sample result was obtained for 3 wt% of the extract. The value of a_tU_ of this sample after the aging process was 142.1 MPa, and was not only higher than the value of the aged R sample, but also higher by ~7 MPa than the value of the un-aged sample containing this amount of extract.

Analysis of the obtained results shows that even with a small content of the tested extracts it is possible to improve the stability of the impact tensile strength during aging. The obtained results are not only better than the results obtained for pure polymer but also for a polymer containing a reference anti-aging compound.

## 4. Conclusions

The main purpose of the presented research was to determine the anti-aging effectiveness of the proposed extracts. The effectiveness of these compounds was determined based on changes in selected properties of polylactide as a function of aging time. Due to the planned application of new materials, it was important to compare their properties with a reference material containing a commonly used commercial anti-aging compound.

Adding coffee, cocoa, or cinnamon extracts to the pure polylactide did not cause difficulties in the production of tested materials. Obtained composites could be processed with parameters typical for this polymer. At small amounts, the tested extracts did not significantly affect the deterioration of selected mechanical and thermal properties of the composites.

The use of small amounts of the tested extracts positively influenced the stability of the determined properties during the aging process. Compared to the pure polylactide subjected to the aging process, the low contents of the test extracts limited the increase in the melt flow rate, the decrease of the degradation temperature, as well as glass transition temperature. The effectiveness of these extracts was comparable to or better than the effectiveness of the reference anti-aging compound.

The aging process also affected the mechanical properties of the tested materials. The largest reduction was observed for pure polylactide. The use of small amounts of extracts positively influenced the stability of tensile strength and impact tensile strength of samples subjected to the process of accelerated aging. The tested extracts inhibited the loss of mechanical strength observed in the case of a pure polylactide sample, and their effectiveness was better than that of the reference anti-aging compound.

It can therefore be concluded that the proposed plant extracts have anti-aging properties in relation to polylactide. Their effectiveness, especially at lower contents, is comparable or better than the effectiveness of the reference anti-aging compound. This creates the possibility of industrial application, especially in the production of products that can be called completely environmentally friendly. They can be successfully used in the production of biodegradable disposable dishes and cutlery, straws, ear buds, sticks, garbage bags, etc. In all these applications, transparency is not required, and the possibility of biodegradation of such products, which very often are a heavy burden for environment, will be a big advantage.

## Figures and Tables

**Table 1:** The test results of melt flow rate (MFR).

	Extract Content [wt%]	MFR [g/10 min]			
		Aging Time [h]			
		0	720	1440	2160
**P**	-	5.5 ± 0.7	5.7 ± 0.7	21.5 ± 1.0	42.5 ± 1.6
**Coffee extract**	0.5	18.5 ± 5.1	24.7 ± 6.9	51.8 ± 5.3	117.9 ± 6.3
	1	37.2 ± 4.0	40.9 ± 10.4	85.0 ± 20.5	138.3 ± 22.0
	3	51.9 ± 2.3	60.1 ± 11.3	99.6 ± 12.7	202.1 ± 33.3
	5	61.5 ± 5.0	61.9 ± 9.14	121.7 ± 15.2	156.7 ± 16.0
	10	71.9 ± 7.0	73.5 ± 21.4	122.9 ± 26.1	177.2 ± 26.6
**Cocoa extract**	0.5	4.9 ± 1.0	7.9 ± 1.1	8.9 ± 0.6	16.3 ± 3.1
	1	6.7 ± 1.4	9.4 ± 2.0	15.0 ± 1.5	15.7 ± 1.5
	3	11.2 ± 3.5	17.6 ± 3.1	27.9 ± 5.0	27.8 ± 3.9
	5	13.8 ± 3.6	22.8 ± 6.0	33.8 ± 8.0	33.0 ± 8.9
	10	19.9 ± 5.1	33.7 ± 5.4	33.5 ± 6.8	48.3 ± 7.8
**Cinnamon extract**	0.5	4.3 ± 0.7	9.9 ± 2.8	14.2 ± 1.4	38.7 ± 2.9
	1	4.2 ± 0.3	8.4 ± 1.7	11.1 ± 1.5	25.5 ± 1.3
	3	5.3 ± 1.2	15.2 ± 3.4	14.3 ± 2.8	32.1 ± 2.9
	5	7.4 ± 1.9	15.4 ± 4.2	22.1 ± 4.6	39.3 ± 7.9
	10	8.6 ± 1.3	22.7 ± 3.5	27.5 ± 3.1	43.4 ± 4.2
**R**	-	4.7 ± 0.6	8.6 ± 1.1	17.8 ± 2.2	36.3 ± 3.2

**Table 2:** Thermogravimetric test results.

	Extract Content [wt%]	T_5%_ [°C]			
		Aging Time [h]			
		0	720	1440	2160
P	-	320.6	300.7	289.9	279.3
Coffee extract	0.5	297.8	288.6	285.5	266.9
	1	273.5	275.0	282.4	287.1
	3	249.7	258.6	253.6	263.9
	5	245.8	249.5	251.3	251.9
	10	240.42	243.9	241.1	239.0
Cocoa extract	0.5	308.9	306.0	286.4	281.7
	1	303.2	307.8	300.3	296.6
	3	295.7	301.3	294.9	282.7
	5	283.3	291.0	286.5	282.9
	10	279.6	280.3	275.4	276.5
Cinnamon extract	0.5	312.62	298.4	298.0	299.0
	1	316.0	320.7	320.7	310
	3	309.27	308.6	315.6	302.3
	5	305.8	301.2	303.1	302.4
	10	277.1	301.8	296.7	289.6
R	-	291.3	299.1	295.1	289.5

**Table 3:** Results of differential scanning calorimetry test.

	Extract Content [wt%]	Aging Time [h]							
		0		720		1440		2160	
		Tg_1_ [°C]	Tg_2_ [°C]	Tg_1_ [°C]	Tg_2_ [°C]	Tg_1_ [°C]	Tg_2_ [°C]	Tg_1_ [°C]	Tg_2_ [°C]
P	-	62.0	60.1	61.4	57.8	60.3	57.0	59.3	55.5
Coffee extract	0.5	61.0	59.8	60.1	59.4	59.7	58.9	62.4	58.7
	1	61.2	59.9	60.4	59.4	59.7	58.8	61.8	57.9
	3	59.9	59.1	59.9	58.8	58.2	57.9	61.1	56.9
	5	59.6	58.5	60.2	58.4	58.5	58.0	60.9	56.9
	10	58.4	58.1	58.6	57.8	58.0	56.6	61.1	55.7
Cocoa extract	0.5	62.9	60.1	62.8	60.0	62.5	59.8	63.2	59.7
	1	62.7	59.5	62.8	60.0	62.7	59.5	62.9	59.9
	3	62.2	59.6	62.9	59.5	62.0	59.2	62.3	59.0
	5	62.1	58.7	62.2	59.0	61.6	58.9	62.2	58.5
	10	61.2	58.6	61.7	58.7	61.2	58.5	62.4	57.9
Cinnamon extract	0.5	62.6	59.7	62.8	59.9	62.4	59.9	62.9	59.3
	1	62.5	59.4	62.7	60.2	61.9	59.7	62.4	59.4
	3	61.6	59.6	61.9	59.6	61.4	59.6	62.2	59.0
	5	60.7	59.9	61.8	59.5	61.7	59.2	62.2	58.9
	10	60.4	59.6	61.3	58.8	61.8	59.1	62.0	58.2
R	-	60.5	59.9	59.9	59.9	60.4	59.6	60.0	57.1

**Table 4:** Results of the tensile test.

	Extract Content [wt%]	Aging Time [h]							
		0		720		1440		2160	
		σ_M_ [MPa]	ε_B_ [%]	σ_M_ [MPa]	ε_B_ [%]	σ_M_ [MPa]	ε_B_ [%]	σ_M_ [MPa]	ε_B_ [%]
P	-	62.2 ± 2.0	12.4 ± 0.6	66.4 ± 2.7	7.0 ± 2.2	59.6 ± 2.0	6.9 ± 1.2	45.5 ± 2.2	6.4 ± 0.7
Coffee extract	0.5	62.6 ± 1.4	11.3 ± 0.7	70.7 ± 1.6	6.5 ± 0.4	66.5 ± 1.7	5.7 ± 0.6	64.2 ± 1.4	5.5 ± 1.0
	1	61.0 ± 1.2	10.7 ± 0.3	71.6 ± 3.1	6.7 ± 0.6	62.6 ± 3.3	5.8 ± 0.5	57.4 ± 4.9	4.7 ± 1.3
	3	61.6 ± 1.2	10.5 ± 0.8	67.2 ± 2.9	6.6 ± 0.4	60.2 ± 3.0	5.2 ± 0.3	56.7 ± 2.7	5.7 ± 0.5
	5	60.7 ± 0.9	10.4 ± 0.4	63.7 ± 2.9	6.7 ± 0.4	56.8 ± 3.0	5.9 ± 0.3	54.0 ± 2.0	5.1 ± 0.7
	10	54.1 ± 1.5	7.9 ± 0.4	56.3 ± 2.0	8.0 ± 1.7	49.9 ± 1.5	6.7 ± 0.6	47.7 ± 1.3	7.5 ± 0.9
Cocoa extract	0.5	61.5 ± 1.9	11.0 ± 0.4	69.3 ± 1.7	7.0 ± 1.1	64.3 ± 1.1	7.2 ± 1.9	61.3 ± 1.6	6.7 ± 1.3
	1	59.4 ± 1.8	9.2 ± 0.8	62.5 ± 4.2	6.6 ± 1.0	60.7 ± 1.8	7.3 ± 1.5	61.5 ± 1.8	7.4 ± 1.2
	3	58.3 ± 1.1	6.9 ± 0.4	61.5 ± 2.2	7.3 ± 1.0	56.8 ± 0.6	7.5 ± 1.4	58.0 ± 1.0	6.3 ± 0.8
	5	52.6 ± 2.1	6.1 ± 0.4	52.2 ± 2.5	5.5 ± 0.5	49.8 ± 2.1	5.6 ± 0.5	50.0 ± 2.0	6.3 ± 0.9
	10	43.1 ± 2.2	4.6 ± 0.6	41.1 ± 1.5	5.0 ± 0.5	37.6 ± 2.3	5.2 ± 0.7	36.5 ± 1.5	5.3 ± 0.5
Cinnamon extract	0.5	59.9 ± 1.7	11.1 ± 0.7	69.1 ± 0.8	8.2 ± 1.0	63.1 ± 1.3	6.9 ± 1.4	59.3 ± 1.3	6.7 ± 1.1
	1	60.6 ± 1.2	11.0 ± 0.7	66.3 ± 2.8	6.8 ± 0.8	60.3 ± 2.7	9.5 ± 2.2	57.3 ± 2.2	7.0 ± 1.2
	3	58.5 ± 1.5	9.9 ± 0.4	63.4 ± 2.8	7.4 ± 1.1	55.7 ± 2.6	9.3 ± 1.1	54.0 ± 2.9	13.3 ± 2.1
	5	57.4 ± 1.6	9.2 ± 0.3	60.0 ± 1.4	7.6 ± 1.2	56.7 ± 2.1	8.3 ± 2.7	53.6 ± 1.0	7.6 ± 1.0
	10	57.7 ± 1.6	6.6 ± 0.5	58.0 ± 2.0	9.8 ± 2.6	52.0 ± 1.4	10.2 ± 1.5	49.5 ± 0.3	14.8 ± 1.7
R	-	55.5 ± 2.1	9.1 ± 0.7	66.4 ± 2.1	6.6 ± 0.6	66.8 ± 1.3	4.8 ± 0.4	61.8 ± 2.1	4.8 ± 0.4

**Table 5:** Results of impact tensile strength (a_tU_) test.

	Extract Content [wt%]	a_tU_ [kJ/m^2^]			
		Aging Time [h]			
		0	720	1440	2160
P	-	140.3 ± 3.4	150.3 ± 11.9	125.3 ± 14.9	112.4 ± 10.7
Coffee extract	0.5	140.5 ± 3.8	145.6 ± 11.9	136.3 ± 13.5	133.0 ± 10.9
	1	140.4 ± 3.6	134.9 ± 8.4	138.4 ± 12.4	90.5 ± 4.1
	3	140.2 ± 3.6	147.6 ± 8.5	120.2 ± 5.6	121.0 ± 8.6
	5	141.0 ± 4.4	151.7 ± 10.7	103.7 ± 6.2	103.9 ± 11.5
	10	126.7 ± 4.3	143.5 ± 10.6	109.4 ± 9.9	109.0 ± 9.6
Cocoa extract	0.5	139.9 ± 3.5	154.2 ± 9.8	135.0 ± 3.3	125.1 ± 10.7
	1	140.8 ± 3.6	160.3 ± 9.2	119.7 ± 9.7	126.3 ± 6.6
	3	131.5 ± 3.3	139.7 ± 14.1	128.7 ± 11.3	123.6 ± 8.1
	5	118.4 ± 3.2	139.1 ± 9.0	117.3 ± 5.3	119.1 ± 13.1
	10	107.9 ± 3.4	148.2 ± 10.0	94.9 ± 2.6	94.7 ± 7.4
Cinnamon extract	0.5	134.0 ± 3.4	145.9 ± 7.9	125.1 ± 9.7	133.8 ± 11.6
	1	133.8 ± 3.6	163.9 ± 10.9	137.5 ± 18.6	121.3 ± 3.3
	3	134.9 ± 3.9	155.0 ± 6.7	145.7 ± 7.3	142.1 ± 11.7
	5	131.9 ± 4.2	168.5 ± 10.8	131.7 ± 11.4	132.4 ± 8.7
	10	130.5 ± 3.8	152.2 ± 4.7	130.6 ± 12.5	126.6 ± 6.9
R	-	145.7 ± 3.9	157.8 ± 14.9	134.7 ± 7.2	118.4 ± 5.4
